# Clinical characteristics of rheumatoid arthritis patients with interstitial lung disease: baseline data of a single-center prospective cohort

**DOI:** 10.1186/s13075-023-03024-8

**Published:** 2023-03-17

**Authors:** Hyoungyoung Kim, Soo-Kyung Cho, Yeo-Jin Song, Juyeon Kang, Seung-A Jeong, Hye Won Kim, Chan-Bum Choi, Tae-Hwan Kim, Jae-Bum Jun, Sang-Cheol Bae, Dae-Hyun Yoo, Hyun Lee, Dong Won Park, Jang Won Sohn, Ho Joo Yoon, Su Jin Hong, Seung-Jin Yoo, Yo Won Choi, Youkyung Lee, Sang Heon Kim, Yoon-Kyoung Sung

**Affiliations:** 1 Department of Rheumatology, Hanyang University Hospital for Rheumatic Diseases, Seoul, Republic of Korea; 2 Hanyang University Institute for Rheumatology Research, Seoul, Republic of Korea; 3 Department of Internal Medicine, Inje University Busan Paik Hospital, Inje University College of Medicine, Busan, Republic of Korea; 4 Department of Internal Medicine, Hanyang University College of Medicine, Seoul, Republic of Korea; 5 Department of Radiology, Hanyang University College of Medicine, Hanyang University Guri Hospital, Guri, Republic of Korea; 6 Department of Radiology, Hanyang University College of Medicine, Hanyang University Hospital, Seoul, Republic of Korea

**Keywords:** Rheumatoid arthritis, Interstitial lung disease, Clinical characteristics, Cohort

## Abstract

**Background:**

To introduce a prospective cohort for rheumatoid arthritis (RA) patients with interstitial lung disease (ILD) and to identify their clinical features in comparison with RA patients without ILD.

**Methods:**

Using a multidisciplinary collaborative approach, a single-center cohort for RA patients with ILD (RA-ILD) was established in May 2017, and enrolment data from May 2017 to March 2021 were used to compare the clinical features of RA patients without ILD (RA-non ILD). Multivariable logistic regression analysis was used to identify factors associated with ILD in RA patients.

**Results:**

Among 148 RA-ILD and 410 RA-non ILD patients, participants in the RA-ILD group were older (65.8 ± 9.9 vs. 58.0 ± 10.4 years, *P* < 0.001) and included more males (35.8% vs. 14.6%, *P* < 0.001) than in the RA-non ILD group. The RA-ILD group had a higher proportion of late-onset RA patients (age ≥ 60 years) than in the comparator group (43.9% vs. 14.2%, *P* < 0.001). Multivariable logistic regression analysis showed that higher age at RA onset (OR 1.056, 95% CI 1.021–1.091), higher body mass index (BMI; OR 1.65, 95% CI 1.036–2.629), smoking history (OR 2.484, 95% CI 1.071–5.764), and oral glucocorticoid use (OR 3.562, 95% CI 2.160–5.874) were associated with ILD in RA patients, whereas methotrexate use was less likely to be associated with ILD (OR 0.253, 95% CI 0.155–0.412).

**Conclusions:**

Higher age at RA onset, smoking history, and higher BMI were associated with the presence of ILD among RA patients. Oral glucocorticoids were more frequently used whereas methotrexate was less likely to be used in RA-ILD patients.

**Supplementary Information:**

The online version contains supplementary material available at 10.1186/s13075-023-03024-8.

## Background

Rheumatoid arthritis (RA) is an autoimmune disease that predominantly affects the musculoskeletal system and leads to significant joint destruction [1]. Interstitial lung disease (ILD) is one of the most common extra-articular manifestations of RA, and RA-ILD is associated with significant morbidity and mortality [2–4]. The prevalence of ILD among RA patients is 1–67.3% and varies by the study design, study population, and definition of ILD [2, 4–10]. One of the challenges in treating RA-ILD is that many of the therapeutic options for RA, such as conventional synthetic disease-modifying anti-rheumatic drugs (csDMARDs) and biologic agents potentially induce pulmonary toxicity [11–15]. Although csDMARDs and biologic agents are widely used for RA-related manifestations in joints, their potential therapeutic benefits for RA-ILD remain controversial [16–20]. Therefore, joint and lung involvement should be evaluated independently of each other for treatment-related decision-making. However, some similarities between RA-ILD and idiopathic pulmonary fibrosis and the recent clinical trials suggest a possible future role for treatment with antifibrotic agents [21].

ILD usually appears after symptomatic joint involvement, but sometimes it might precede the articular manifestations [22]. As the clinical manifestations usually appear in only advanced-stage lung disease, early diagnosis of RA-ILD proves challenging. Furthermore, the progression and the severity of lung involvement are the two major factors to consider for treatment-related decision-making. Though dyspnea and cough are the most common symptoms in patients with ILD, up to 30% of RA patients with or without respiratory symptoms had subclinical ILD on high-resolution computerized tomography (HRCT) scanning of the chest [23, 24]. Due to the large variation in the clinical course of ILD in RA patients, ILD is difficult to diagnose without a radiologic evaluation in this population. The diagnostic approach to patients with ILD in the setting of known or suspected RA requires a collaborative multidisciplinary approach, with expert radiology, rheumatology, and pulmonology inputs, whereas evaluating for other potential causes of ILD, such as hypersensitivity pneumonitis, pneumoconiosis, connective tissue diseases (CTD) other than RA, or iatrogenic causes, such as drug toxicity [25].

This study was conducted to introduce a Korean RA-ILD registry that was established with a multidisciplinary collaborative approach with an emphasis on the study design and, by using the baseline data, to provide clinical characteristics of the RA-ILD patients who are currently registered in this prospective cohort.

## Materials and methods

### Establishment of a prospective cohort for RA-ILD patients

#### Study purpose and outcomes

We designed and established a prospective cohort study of RA patients at a single center to compare the long-term prognosis between RA-ILD patients and RA patients without ILD (RA-non ILD) and to explore prognostic factors among RA-ILD patients.

#### Study population

**Fig. 1:**
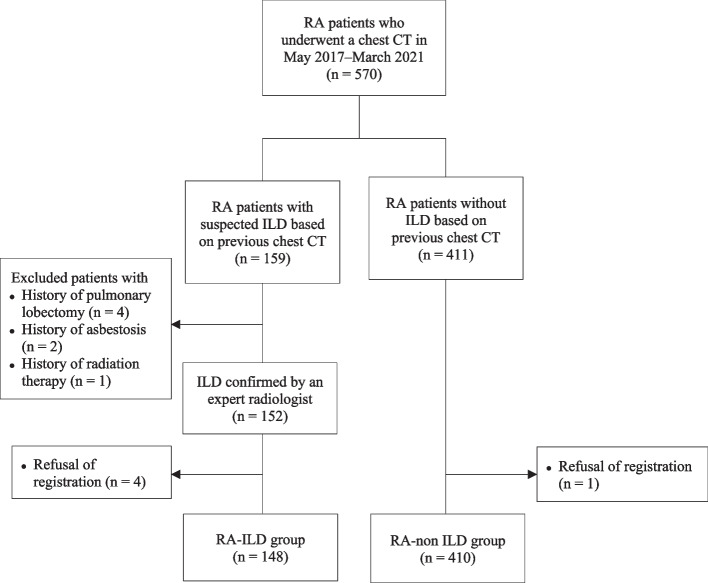
Patient selection flow in this study. RA, rheumatoid arthritis; ILD, interstitial lung disease; CT, computed tomography

Patients were eligible for inclusion if they were 19 years or older, met the 1987 American College of Rheumatology (ACR) [26] or 2010 American College of Rheumatology/European League Against Rheumatism (ACR/EULAR) classification criteria for RA [27], underwent a chest CT scan within the 2 years preceding enrolment, and provided written consent to participate in this study. The RA-ILD group was selected based on the chest CT scan after re-evaluation by radiologists and rheumatologists if they had interstitial lung abnormalities that were indicative of RA-ILD. We excluded patients in whom it was difficult to evaluate the extent and type of ILD, such as those with a history of radiation therapy (*n* = 1), asbestosis (*n* = 2), or pulmonary lobectomy (*n* = 4). Among the RA patients who underwent CT within the previous 2 years, only those who did not have ILD or other pulmonary comorbidities, such as malignancy, pneumonia, or active pulmonary tuberculosis, were included in the RA-non ILD group (Fig. 1).

#### Enrolment and follow-up

After enrolment, the demographics and clinical information were collected, and the RA disease activity was evaluated by rheumatologists. We assessed the functional disability and quality of life by using a self-reported questionnaire that was completed by the participants with the assistance of a well-trained healthcare professional. We planned to conduct annual follow-up with patients and to collect their comprehensive clinical information. The underlying lung function of patients with ILD at the time of enrolment was assessed through a pulmonary function test (PFT) and echocardiography, if possible (Additional Table 1), and the performance of those tests could be adjusted according to the clinical situation of each patient and were scheduled at every 2-year follow-up visit.

### Clinical characteristics and associated factors of RA-ILD patients

We collected data on demographic features, such as age at enrolment, age at RA diagnosis, sex, body mass index (BMI), smoking, income, and education level; clinical characteristics, such as disease activity score in 28 joints (DAS28) based on the erythrocyte sedimentation rate (ESR) or CPR (C-reactive protein) and comorbidities based on the Charlson Comorbidity Index, the Health Assessment Questionnaire-Disability Index (HAQ-DI), and the EuroQol-5 Dimension Index (EQ-5D); laboratory tests, including rheumatoid factor (RF), anti-citrullinated peptide antibody (ACPA), ESR, and CRP; and medication use, including DMARDs and glucocorticoid. In this study, we compared the demographics and clinical characteristics at enrolment between RA-ILD and RA-non ILD patients.

### Statistical analysis

Categorical variables are presented as numbers and percentages (%), and continuous variables are presented as means with standard deviations (SD). We employed the independent *t*-test and the chi-square test to compare the two groups. Factors related to the presence of ILD among RA patients were analyzed using multivariable logistic regression analysis. All analyses were performed using SAS® 9.4 (SAS Institute, Cary, NC, USA), and results were considered statistically significant when *P*-values were less than 0.05.

## Results

### Demographic and clinical characteristics of RA-ILD and RA-non ILD patients

In total, 148 RA-ILD and 410 RA-non ILD patients were enrolled between May 2017 and March 2021 (Fig. 1). The mean age at enrolment and at RA diagnosis was significantly higher in the RA-ILD group than in the RA-non ILD group (65.8 ± 9.9 vs. 58.0 ± 10.4 and 57.4 ± 11.3 vs. 47.1 ± 12.0, respectively; *P* < 0.001 for both). The disease duration of ILD of RA-ILD group was 2.1 ± 1.8 years on average. The proportion of patients with late-onset RA (age > 60 years) was higher in the RA-ILD group than in the comparator (43.9% vs. 14.2%, *P* < 0.001). The RA-ILD group included more male patients (35.8 vs. 14.5%, *P* < 0.001) and comprised more smokers compared to the RA-non ILD group (38.5% vs. 18.5%, *P* < 0.001). The RF positivity was higher in the RA-ILD group compared to that in the RA-non ILD group whereas the prevalence of anti-CCP antibody was comparable between the two groups. At enrolment, the disease activity as estimated by DAS28-ESR was significantly higher in the RA-ILD group (3.7 ± 1.4 vs. 3.4 ± 1.2, *P* = 0.007). Inflammatory markers were elevated more in the RA-ILD group than in the comparators (ESR 38.5 ± 27.5 vs. 26.5 ± 20.4 mm/h, *P* < 0.001 and CRP 1.0 ± 1.8 vs. 0.5 ± 0.8 mg/dL, *P* < 0.001; Table 1).

**Table 1:** Clinical characteristics of patients with and without ILD

**Variables**	**RA-ILD**	**RA-non ILD**	***P***
	**(** ***n*** ** = 148)**	**(** ***n*** ** = 410)**	
Sex, female	95 (64.2)	350 (85.4)	< 0.001
Age, years	65.8 ± 9.9	58.0 ± 10.4	< 0.001
BMI (*n* = 74, 250)	23.6 ± 3.5	23.0 ± 3.2	0.055
BMI < 23 kg/m^2^	61 (41.2)	201 (49.0)	0.103
BMI ≥ 23 kg/m^2^	87 (58.8)	209 (51.0)	
Smoking history			
Never smoker	91 (61.5)	336 (82.0)	< 0.001
Ever smoker	57 (38.5)	74 (18.0)	
Pack-years of cigarettes smoked (*n* = 57, 74)	31.0 ± 24.6	21.7 ± 16.2	0.018
CCI score^a^	0 (0, 1)	0 (0, 0)	< 0.001
Disease duration of RA-ILD, years	2.1 ± 1.8	N/A	N/A
ILD on chest X-ray	107 (72.3)	N/A	N/A
Age of RA diagnosis, years	57.4 ± 11.3	47.1 ± 12.0	< 0.001
Late-onset RA	65 (43.9)	58 (14.2)	< 0.001
Younger-onset RA	83 (56.1)	352 (85.9)	
Disease duration of RA, years	8.4 ± 7.0	10.9 ± 7.5	< 0.001
Disease activity of RA			
DAS28-ESR	3.7 ± 1.4	3.4 ± 1.2	0.007
DAS28-CRP	2.5 ± 1.1	2.4 ± 1.0	0.234
CDAI	10.9 ± 8.9	9.9 ± 7.3	0.358
SDAI	11.9 ± 9.9	10.3 ± 7.6	0.156
Quality of life			
HAQ-DI	0.9 ± 0.7	0.7 ± 0.6	0.002
HAQ-DI ≥1	66 (44.6)	127 (31.0)	< 0.001
HAQ-DI <1	82 (55.4)	283 (69.0)	
EQ-5D	0.7 ± 0.2	0.8 ± 0.1	< 0.001
Laboratory test			
RF positivity	132 (89.2)	330 (80.5)	0.016
Anti-CCP positivity (*n* = 145, 410)	134 (91.8)	373 (91.2)	0.830
Either RF or anti-CCP positivity	141 (95.3)	397 (96.8)	0.382
ESR, mm/hr	38.5 ± 27.5	26.5 ± 20.4	< 0.001
CRP, mg/dL	1.0 ± 1.8	0.5 ± 0.8	< 0.001
Medications			
Conventional synthetic DMARDs			
Methotrexate	80 (54.1)	339 (82.7)	< 0.001
Sulfasalazine	60 (40.5)	75 (18.3)	< 0.001
Hydroxychloroquine	63 (42.6)	74 (18.1)	< 0.001
Leflunomide	8 (5.4)	100 (24.4)	< 0.001
Tacrolimus	24 (16.2)	39 (9.5)	0.027
Biologic DMARDs	25 (16.9)	83 (20.2)	0.376
TNF inhibitors^b^	9 (6.1)	65 (15.9)	0.003
Non-TNF inhibitors^c^	16 (10.8)	18 (4.4)	0.005
Targeted synthetic DMARD^d^	1 (0.7)	14 (3.4)	0.133
Oral glucocorticoids	81 (54.7)	96 (23.4)	< 0.001
Glucocorticoids dose^e^, mg/day	4.9 ± 3.1	3.0 ± 1.6	< 0.001
Anti-fibrotic agents^f^	1 (0.7)	N/A	N/A

Data are presented as numbers with percentages or mean with standard deviation

*Anti-CCP antibody* Anti-citrullinated peptide antibody, *BMI* body mass index, *CRP* C-reactive protein, *DAS28* Disease Activity Score in 28 joints, *DMARD* disease-modifying anti-rheumatic drug, *ESR* erythrocyte sedimentation rate, *ILD* interstitial lung disease, *RA* rheumatoid arthritis, *RF* rheumatoid factor, *TNF* tumor necrosis factor, *N/A* not applicable

^a^All patients had rheumatoid arthritis, and thus connective tissue diseases were excluded from the calculation of CCI in this study

^b^TNF blockers include adalimumab, etanercept, golimumab, and infliximab. In the RA-ILD group, 7 patients used adalimumab, 1 used etanercept, and 1 used golimumab; in the RA-non-ILD group, 25 patients used adalimumab, 30 used etanercept, 9 used golimumab, and 1 used infliximab

^c^Non-TNF blockers include abatacept, tocilizumab, and rituximab. In the RA-ILD group, 11 patients used abatacept, 3 used tocilizumab, and 2 used rituximab; in the RA-non-ILD group, 8 patients used abatacept, 7 used tocilizumab, and 3 used rituximab

^d^Targeted synthetic DMARDs include tofacitinib

^e^Regularly prescribed oral glucocorticoids were considered and the average was presented as a prednisone-equivalent dose

^f^One RA-ILD patient was using nintedanib at the time of enrolment

The presence of ILD among RA patients may affect their medication use for controlling the RA disease activity. Table 1 shows that oral glucocorticoids were used more frequently in the RA-ILD group (54.7 vs. 23.4%, *P* < 0.001), with an approximately 1.4-times higher daily dose compared to the RA-non ILD group. The use of MTX and leflunomide was less frequent in the RA-ILD group (54.1% vs. 82.7% and 5.4% vs. 24.4%, respectively; *P* < 0.001 for both), whereas hydroxychloroquine and sulfasalazine were more frequently used in the RA-ILD group than in the RA-non ILD group (42.6 vs. 18.1% and 40.5 vs. 18.3%, respectively, *P* < 0.001 for both). Despite a difference in the use of each agent, there was no significant difference in the use of biological agents between the two groups (16.9 vs. 20.2%, *P* = 0.376); non-tumor necrosis factor (TNF) inhibitors were used more frequently in the RA-ILD group (10.8% vs. 4.4%, *P* = 0.005), whereas TNF-inhibitor use was more prevalent in the RA-non ILD group (6.1% vs. 15.9%, *P* = 0.003).

### Factors related to the presence of ILD among RA patients

In a multivariable analysis, a higher BMI (OR 1.65, 95% CI 1.036–2.629), history of smoking (OR 2.484, 95% CI 1.071–5.764), and higher age at RA diagnosis (OR 1.056, 95% CI 1.021–1.091) were significantly associated with ILD in RA patients. Oral glucocorticoid use was more likely in RA-ILD patients (OR 3.853, 95% CI 2.32–6.4), whereas MTX (OR 0.253, 95% CI 0.155–0.412) was less likely to be used in RA-ILD patients (Table 2).

**Table 2:** Factors related to the presence of RA-ILD in RA patients

**Variables**	**Univariable** **(** ***n*** ** = 558)**		**Multivariable** **(** ***n*** ** = 558)**	
	Crude OR (95% CI)	*P*	Adjusted OR (95% CI)	*P*
Age, years	1.082 (1.059, 1.106)	<0.001	1.017 (0.979, 1.056)	0.393
Sex (ref. female)	3.254 (2.110, 5.020)	<0.001	0.969 (0.392, 2.393)	0.945
BMI ≥ 23 kg/m^2^ (ref. BMI < 23 kg/m^2^)	1.372 (0.938, 2.006)	0.103	1.650 (1.036, 2.629)	0.035
Ever-smoker (ref. never-smoker)	2.844 (1.876, 4.311)	<0.001	2.484 (1.071, 5.764)	0.034
Age at RA diagnosis	1.081 (1.060, 1.102)	<0.001	1.056 (1.021, 1.091)	0.001
Late-onset RA (ref. younger-onset RA)	4.753 (3.100, 7.286)	<0.001		
Disease duration of RA	0.949 (0.921, 0.977)	0.001		
RF positivity (ref. RF negative)	2.000 (1.127, 3.549)	0.018	1.222 (0.630, 2.369)	0.552
Anti-CCP positivity (ref. Anti-CCP negative) (*n* = 554)	1.070 (0.540, 2.117)	0.847		
Either RF or anti-CCP positivity (*n* = 554)	0.659 (0.258, 1.686)	0.385		
ESR	1.021 (1.013, 1.030)	<0.001		
CRP	1.489 (1.239, 1.790)	<0.001		
DAS28-ESR	1.244 (1.074, 1.440)	0.004	0.952 (0.739, 1.228)	0.706
DAS28-CRP	1.161 (0.970, 1.391)	0.104		
CDAI	1.017 (0.993, 1.041)	0.166		
SDAI	1.022 (1.000, 1.044)	0.052		
HAQ ≥ 1 (ref. HAQ <1)	1.794 (1.220, 2.638)	0.003	1.294 (0.768, 2.179)	0.333
Methotrexate use (ref. not used)	0.246 (0.163, 0.372)	<0.001	0.251 (0.154, 0.410)	<0.001
Biologic DMARDs use (ref. not used)	0.688 (0.425, 1.112)	0.127		
Oral Glucocorticoid use (ref. not used)	3.954 (2.661, 5.877)	<0.001	3.853 (2.320, 6.400)	<0.001

We included independent variables in the multivariable analysis; variables with *P < *0.1 in the univariable analysis were included, and multicollinear explanatory variables were excluded

*Anti-CCP antibody* anti-citrullinated peptide antibody, *BMI* body mass index, *CRP* C-reactive protein, *DAS28* Disease Activity Score in 28 Joints, *DMARD* disease-modifying anti-rheumatic drug, *ESR* erythrocyte sedimentation rate, *EQ-5D* EuroQol-5 Dimension, *HAQ-DI* Health Assessment Questionnaire Disability Index, *ILD* interstitial lung disease, *RA* rheumatoid arthritis, *RF* rheumatoid factor

### Respiratory symptoms and pulmonary function test results of RA-ILD patients

Among the 148 RA-ILD patients who had initial CT, usual interstitial pneumonia (UIP) was the most common subtype of ILD (*n* = 107, 72.8%), followed by non-specific interstitial pneumonia (NSIP; *n* = 35, 23.6%), organizing pneumonia (OP; *n* = 1, 0.7%), and respiratory bronchiolitis-interstitial lung diseases (*n* = 1, 0.7%; Table 3).

**Table 3:** Predominant type of ILD in RA patients

**Variables**	**Total**
	**(** ***n*** ** = 148)**
UIP	107 (72.3)
NSIP	35 (23.6)
OP	1 (0.7)
RB-ILD	1 (0.7)
Undetermined	4 (2.7)

Data are presented as numbers with percentages

*ILD* interstitial lung disease, *LIP* lymphocytic interstitial pneumonia, *NSIP* nonspecific interstitial pneumonia, *OP* organizing pneumonia, *RA* rheumatoid arthritis, *RB-ILD* respiratory bronchiolitis-interstitial lung diseases, *UIP* usual interstitial pneumonia

The most common symptom of ILD was dyspnea, followed by sputum and cough (Supplementary Table 2). The RA patients with ILD in this cohort had relatively well-preserved pulmonary function in accordance with 81.4 ± 15.1% predicted value in forced vital capacity (FVC). A moderate degree of reduction in diffusion capacity was noted in the diffusion capacity of the lungs for carbon monoxide (DLco; 57.1 ± 13.8% predicted). These are summarized in Supplementary Table 2.

## Discussion

Based on a multidisciplinary collaborative approach that was implemented according to a systematic protocol, we established a prospective cohort for RA-ILD patients. In the cross-sectional study which used the enrolment data of this prospective cohort, we demonstrated that higher age at RA diagnosis, a higher BMI, and smoking were associated with accompanying ILD in RA patients. In addition, we found that RA-ILD patients tended to use glucocorticoids more frequently and less frequently used methotrexate as compared with RA-non ILD patients.

There are no definitive guidelines for screening patients with RA for the presence of ILD at present; therefore, screening is usually performed according to the clinical needs of each patient. In our study, though we included definite RA-ILD patients, 28.4% of these participants had no definite respiratory symptoms and 27.3% did not have any indicative findings of ILD on simple chest X-ray. Instead, most RA-non ILD patients were evaluated using a chest CT scan for screening for lung diseases before starting targeted therapies or to identify the underlying pulmonary pathology. According to our previous study, the prevalence of old pulmonary tuberculosis (18.2%) or bronchiectasis (27.5%) in South Korea was relatively high as compared to that in Western countries [28–30]. In addition, our study might suggest that associated factors for RA-ILD, such as a higher BMI, history of smoking, and higher age at RA diagnosis, are worth considering when planning chest CT scan to screen for the presence of ILD in RA patients.

Sometimes, a problem that is faced when studying ILD that it is clinically complex to capture the exact timing of occurrence of ILD because chest CT scanning is usually considered only if a patient is suffering from any respiratory symptoms, rather than for screening purposes. Therefore, many clinical studies could not include patients with subclinical ILD which might affect the natural history of RA. The sequence in which both RA and ILD occurred was uncertain, and RA occurrence was sometimes followed by ILD. In clinical practice, physicians are concerned that ILD might progress or become exacerbated on exposure to certain DMARDs. This concern usually affects the treatment decision-making for RA-ILD patients. Insufficient medication use could result in higher disease activities of RA, and it is hard to assess causality whether a certain drug or uncontrolled RA disease activity affected the occurrence of ILD.

Previous studies identified traditional risk factors for ILD including smoking [18], older age [31], male sex [32], longer RA duration [31], elevated inflammatory markers [33], and the RA serologic status [34]. Except for smoking, these risk factors were non-modifiable. A higher disease activity of RA was associated with an increased risk of developing RA-ILD [35] as well as a poor prognosis for RA-ILD patients [36]. The disease activity of RA was a modifiable risk factor; however, the study population already had longstanding RA at baseline, which might have affected the occurrence of ILD. Though the confounders were adjusted, it is unclear whether RA itself or uncontrolled disease activity was associated with an increased risk of ILD.

In this study, we demonstrated the distinct characteristics of RA-ILD patients in comparison with RA-non ILD patients. The results of our study consistently match those of previous studies which showed that a history of smoking was an associated factor for the presence of ILD among RA patients. Smoking itself may contribute to the citrullination of proteins and induce ACPA by promoting autoimmune responses [37]. However, RF or ACPA positivity was not associated with ILD in RA patients in our study, though previous studies reported that concentrations of RF or ACPA were associated with prevalent RA-ILD [38] or increased the risk of disease progression and mortality [39]. In our study, the positive rate of RF was higher in RA-ILD group compared to RA-non ILD group, but this was not associated with ILD after adjusting confounders. This suggests that, regardless of seropositivity, smoking may be independently associated with ILD. Being male and older were not significant factors whereas the age at RA onset was an independent associated factor for ILD in the multivariable analysis.

Higher BMI was another associated factor for ILD in RA patients in our study. However, it is unclear whether obesity is a cause of ILD development or rather the sequelae of glucocorticoid treatment or limitation of physical activities [40]. Through our prospective cohort study, we expected to investigate whether BMI constitutes an aggravating factor in RA-ILD patients or a risk factor for ILD occurrence in RA-non ILD patients.

With regard to medication, there were apparently different patterns of DMARD use based on the presence of ILD. For example, RA-ILD patients used MTX and leflunomide less frequently but used hydroxychloroquine and sulfasalazine more frequently than those without ILD. Though the causative association between MTX use and ILD development remains inconclusive, which is similar to the conclusion of previous studies [41–43], the presence of ILD could guide clinicians to avoid specific medications for their patients. The use of oral glucocorticoids was related to the presence of RA-ILD in our analysis. This indicates that clinicians tend to choose glucocorticoids to treat both RA and ILD because of concerns with regard to inducing or exacerbating ILD with DMARDs. Using cumulative clinical information, we expect that the results of our prospective cohort study would facilitate an assessment of the relationship between certain medications and the occurrence or progression of ILD.

This study has some limitations. We could not investigate causal relationships between clinical factors and ILD in RA patients because of the cross-sectional study design. Thus, our results are not conclusive. Instead, prospective data based on our cohort which includes RA-non ILD RA patients will provide a better understanding of the natural course of ILD development in RA patients. The advantage of our prospective cohort is the simultaneous enrolment of RA-non ILD patients as comparators based on a multidisciplinary approach.

## Conclusion

In conclusion, the presence of RA-ILD was associated with a higher RA-onset age, smoking history, and a higher BMI. Oral glucocorticoid use was more likely in RA-ILD patients, but MTX was less likely to be used.

## Supplementary Information

**Additional file 1: Supplementary Table 1.** Variables assessed at enrolment and follow-up in the cohort. **Supplementary Table 2.** Pulmonary symptoms and test results of RA-ILD patients.

## Data Availability

The data analyzed in this article cannot be shared publicly owing to the requirements for protecting the privacy and confidentiality of the participants. The research data will be shared on reasonable request made to the corresponding author.

## Abbreviations

RA: Rheumatoid arthritis
ILD: Interstitial lung disease
csDMARDs: Conventional synthetic disease-modifying anti-rheumatic drugs
HRCT: High-resolution computerized tomography
CTD: Connective tissue disease
RA-non ILD: RA patients without ILD
ACR: American College of Rheumatology
EULAR: European League Against Rheumatism
PFT: Pulmonary function test
BMI: Body mass index
DAS28: Disease Activity Score in 28 Joints
ESR: Erythrocyte sedimentation rate
CPR: C-reactive protein
HAQ-DI: Health Assessment Questionnaire-Disability Index
EQ-5D: EuroQol-5 Dimension Index
RF: Rheumatoid factor
SD: Standard deviation
UIP: Interstitial pneumonia
NSIP: Nonspecific interstitial pneumonia
LIP: Lymphocytic interstitial pneumonia
