# Supplementary material for: Clinical characteristics of rheumatoid arthritis patients with interstitial lung disease: baseline data of a single-center prospective cohort
Source: Arthritis Res Ther. 2023 Mar 17;25:43. doi: 10.1186/s13075-023-03024-8 (PMC10022152; doi:10.1186/s13075-023-03024-8)
Supplement: Supplementary file 1 — Additional file 1: Supplementary Table 1. Variables assessed at enrolment and follow-up in the cohort. Supplementary Table 2. Pulmonary symptoms and test results of RA-ILD patients. [file 13075_2023_3024_MOESM1_ESM.docx]

**Additional file 1: Supplementary Tables**

**Supplementary Table 1.** Variables assessed at enrolment and follow-up in the cohort

| **Time (years)** | **Baseline (0)** | **1** | **2** | **3** | **4** |
| --- | --- | --- | --- | --- | --- |
| Informed consent | O | X | X | X | X |
| Demographics | O | O | O | O | O |
| Height, weight, vital signs | O | O | O | O | O |
| RA diagnostic date | O | X | X | X | X |
| History of vaccination | O | X | X | X | X |
| Comorbidities | O | O | O | O | O |
| Laboratory tests | O | O | O | O | O |
| Previous medication use | O | X | X | X | X |
| Current medication use | O | O | O | O | O |
| RA disease activity: DAS28-ESR, DAS28-CRP, SDAI, and CDAI | O | O | O | O | O |
| Patient-reported outcomes: HAQ-DI, EQ-5D | O | O | O | O | O |
| Global VAS health assessment | O | O | O | O | O |
| Chest, hand, and foot X-rays | O | X | o | X | O |
| HRCT^a^ | O | X | X | X | X |
| ILD patients (can be adjusted according to the clinical situation) | | | | | |
| HRCT follow-up |  |  | O |  | O |
| PFT | O | O | O | O | O |
| 6MWT | O | O | O | O | O |
| Echocardiography | O | O | O | O | O |

CDAI, Clinical Disease Activity Index; CRP, C-reactive protein; DAS28, Disease Activity Score in 28 Joints; ESR, erythrocyte sedimentation rate; EQ-5D, EuroQol-5 dimension; HAQ-DI, Health Assessment Questionnaire Disability Index; HRCT, high-resolution computed tomography; ILD, interstitial lung disease; PFT, pulmonary function test; RA, rheumatoid arthritis; SDAI, Simple Disease Activity Index; 6MWT, 6-Minute Walk Test; VAS, visual analog scale

^a^Any previous chest CT in RA-ILD patients and previous chest CT within the preceding 2 years in RA-non-ILD patients.

**Supplementary Table 2.** Pulmonary symptoms and test results of RA-ILD patients

| **Variables** | **RA-ILD**  **(n = 148)** |
| --- | --- |
| Number of patients with any ILD-related symptoms | (71.6) |
| Dyspnoea^a^ | 76 (50.7) |
| Sputum | 59 (39.9) |
| Cough | 57(38.5) |
| Chest pain | 10 (6.8) |
| Weight loss | 9 (6.1) |
| Fever | 5 (3.4) |
| Pulmonary function test (n=147) |  |
| FVC (L) | 2.8 ± 0.7 |
| FVC (% predicted) | 81.4 ± 15.1 |
| FEV1 (L) | 2.2 ± 0.6 |
| FEV1 (% predicted) | 84.2 ± 16.1 |
| FEV1/FVC (% predicted) | 78.0 ± 8.6 |
| Diffusion capacity (n=140) |  |
| DLco (%) | 57.1 ± 13.8 |
| DLco/VA (%) | 69.4 ± 13.6 |

Data are presented as numbers with percentages or as mean with standard deviation.

RA, rheumatoid arthritis; ILD, interstitial lung disease; SD, standard deviation; MMRC, Modified Medical Research Council; FVC, forced vital capacity; FEV1, Forced Expiratory Volume in the first second; DLco, diffusion capacity of the lungs

^a^In total, 58, 13, and 5 patients had dyspnoea of Medical Research Council (MRC) scales 1, 2, and 3, respectively.
